# Supplementary material for: Definition and Discovery of Tandem SH3-Binding Motifs Interacting with Members of the p47phox-Related Protein Family
Source: Biomolecules. 2025 Nov 22;15(12):1641. doi: 10.3390/biom15121641 (PMC12730246; doi:10.3390/biom15121641)
Supplement: Supplementary file 1 [file biomolecules-15-01641-s001.zip › Supplementary Material Figures.pdf]

Definition and discovery of tandem SH3-binding motifs interacting with members of the p47phox-related protein family

Zsofia Etelka Kalman[1], Tamas Lazar[2,3], Laszlo Dobson[4,5,*], Rita Pancsa[4*]

[1] Faculty of Information Technology and Bionics, Pázmány Péter Catholic University, Budapest, Hungary
[2] Structural Biology Brussels (SBB), Department of Bioengineering Sciences, Vrije Universiteit Brussel (VUB), 1050 Brussels, Belgium
[3] VIB-VUB Center for Structural Biology, Vlaams Instituut voor Biotechnologie (VIB), 1050 Brussels, Belgium
[4] Institute of Molecular Life Sciences, HUN-REN Research Centre for Natural Sciences, H-1117, Budapest
[5] Department of Bioinformatics, Semmelweis University, Tűzoltó u. 7, Budapest 1094, Hungary
Correspondence to Rita Pancsa, email: pancsa.rita@ttk.hu or Laszlo Dobson, email: dobson.laszlo@ttk.hu

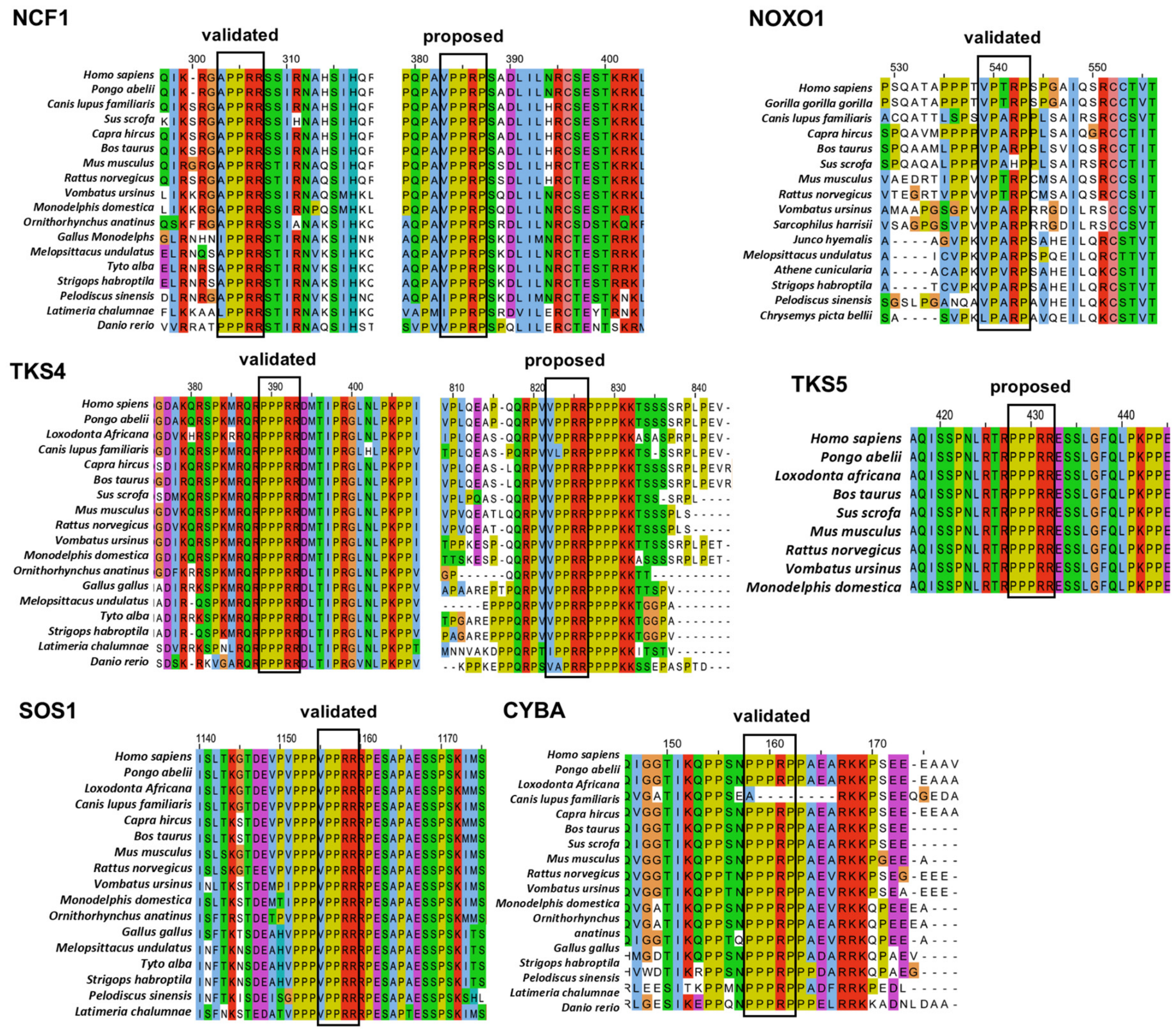

Figure S1: Sequence alignments of the experimentally verified and proposed motifs from Table 1 on vertebrate species. Only the proximity of the motifs are depicted and the motifs themselves are boxed.

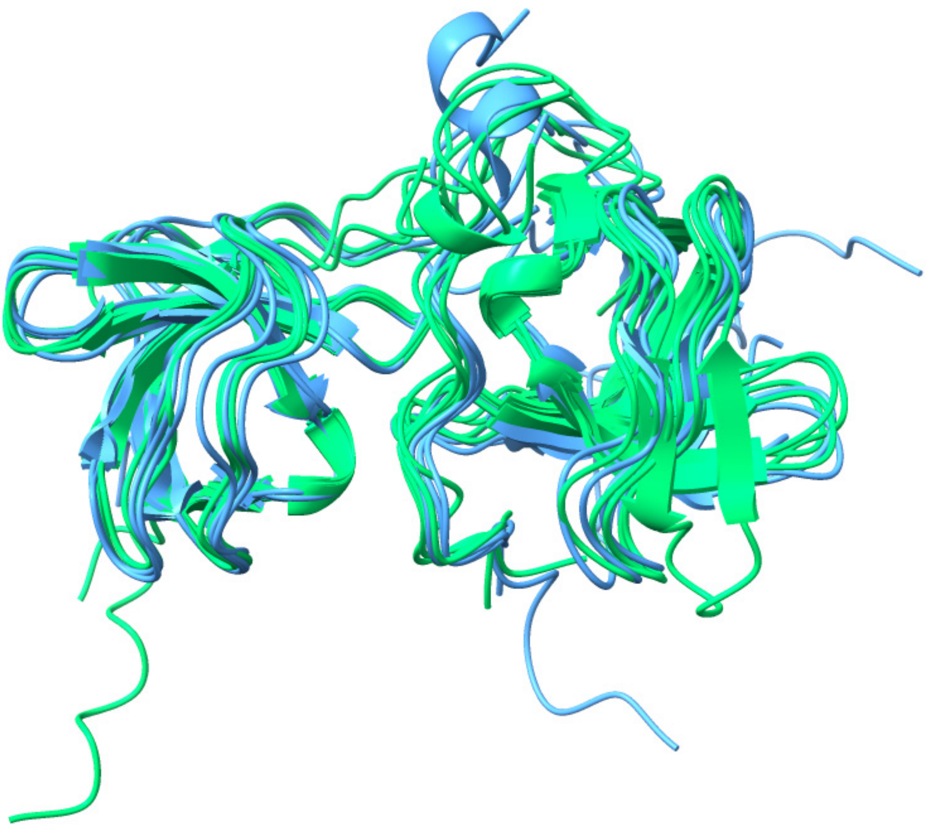

Figure S2: Structure alignment showing the correspondence between experimental and AF2-predicted structures. The experimental structures (1ov3, 1wlp, 7yxw) of tandem SH3 domains with the SH3-binding motifs are depicted in blue, while the AlphaFold2-predicted structures of motifs fitting the strong motif definition are shown in green.

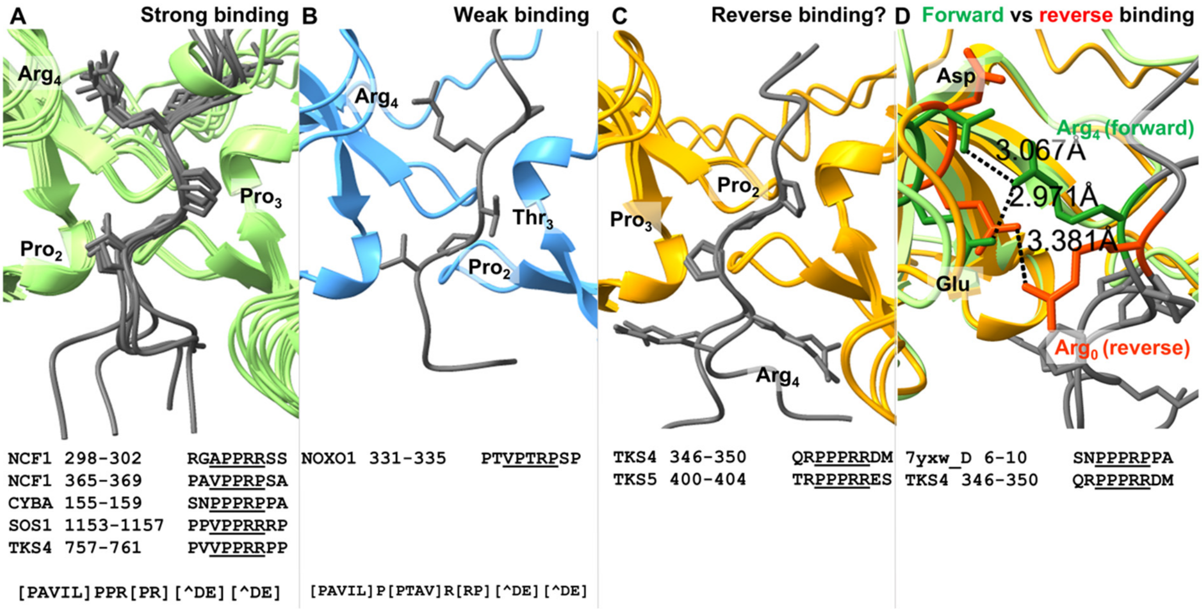

Figure S3: AlphaFold predicts different binding modes for the tandem SH3-binding motifs. AlphaFold-predicted models of the complexes suggest different binding modes based on motif sequence characteristics. A) All motifs fitting the strong motif definition bind very similarly (this is also evident based on the experimentally determined structures listed in **Table 1**). In this version of the Figure the helix following the NCF1 autoinhibitory peptide is shown. B) The weak binder NOXO1 C-terminal motif that has a Thr in the 3rd position of its core binds in an almost identical manner as the strong binders, which supports the validity of the weak motif definition. C) A reverse binding mode is predicted for a pair of homologous motifs in TKS4 and TKS5, where a negatively charged residue follows the core motif, but an Arg occupies position 0, directly preceding the core motif. D) Comparison of the positioning of Arg4 in the forward binding mode versus Arg0 in the reverse binding mode and their distances to the Glu and Asp residues of the C-double negative signature (defined in a following section) on the domain side.

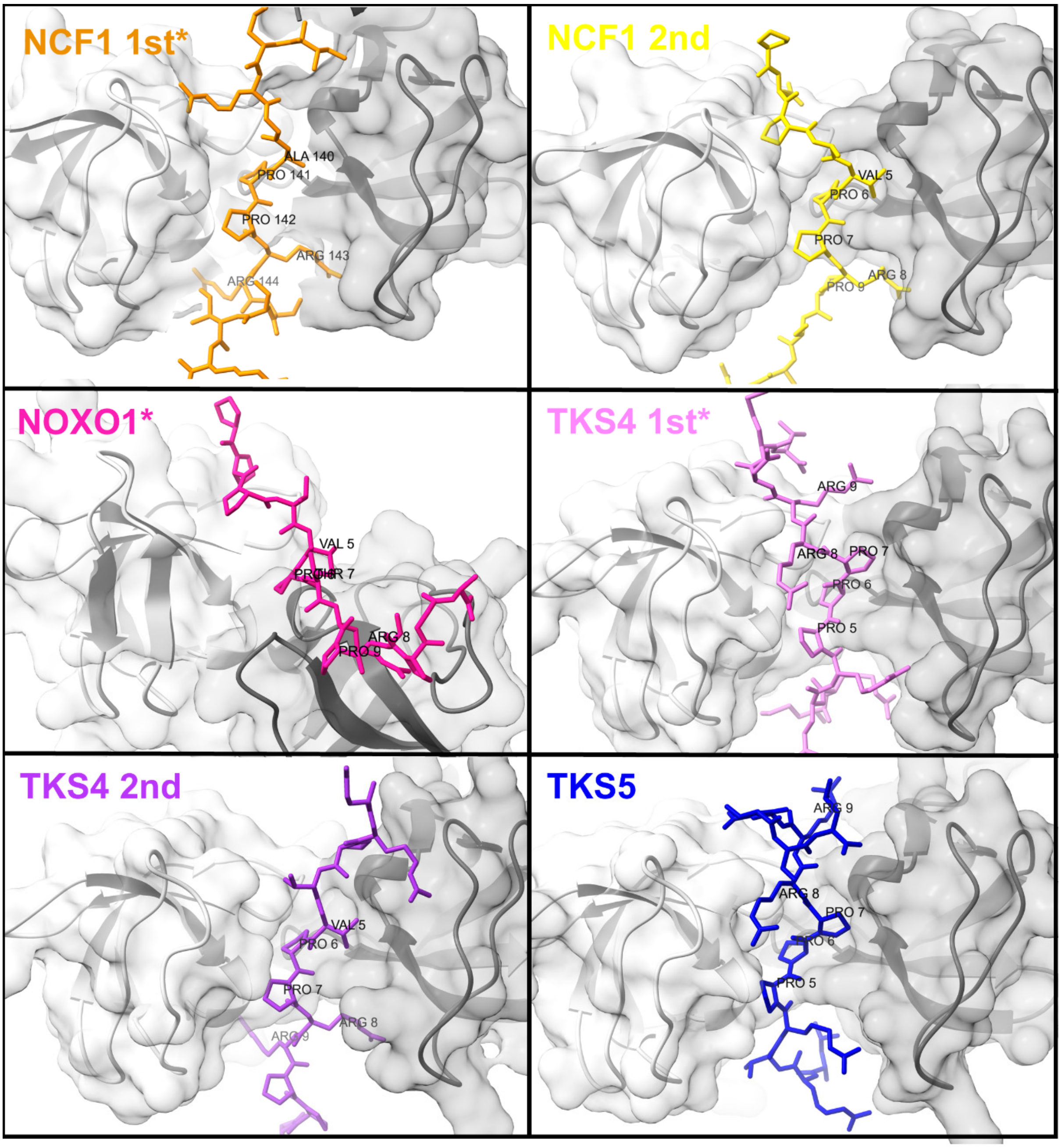

Figure S4: AlphaFold3 models of the tandem SH3 domains with their autoinhibitory peptide(s). The NCF1 1st and 2nd, as well as the TKS4 2nd motifs that fit the strong motif definition bind in forward orientation, while the TKS4 1st and TKS5 motifs bind in reverse orientation (the directions correspond to the ones predicted by AF2). The weak-binding NOXO1 motif is not placed into the same binding groove as the other motifs. The experimentally validated autoregulatory motif instances are marked by a star, the others are proposed instances.

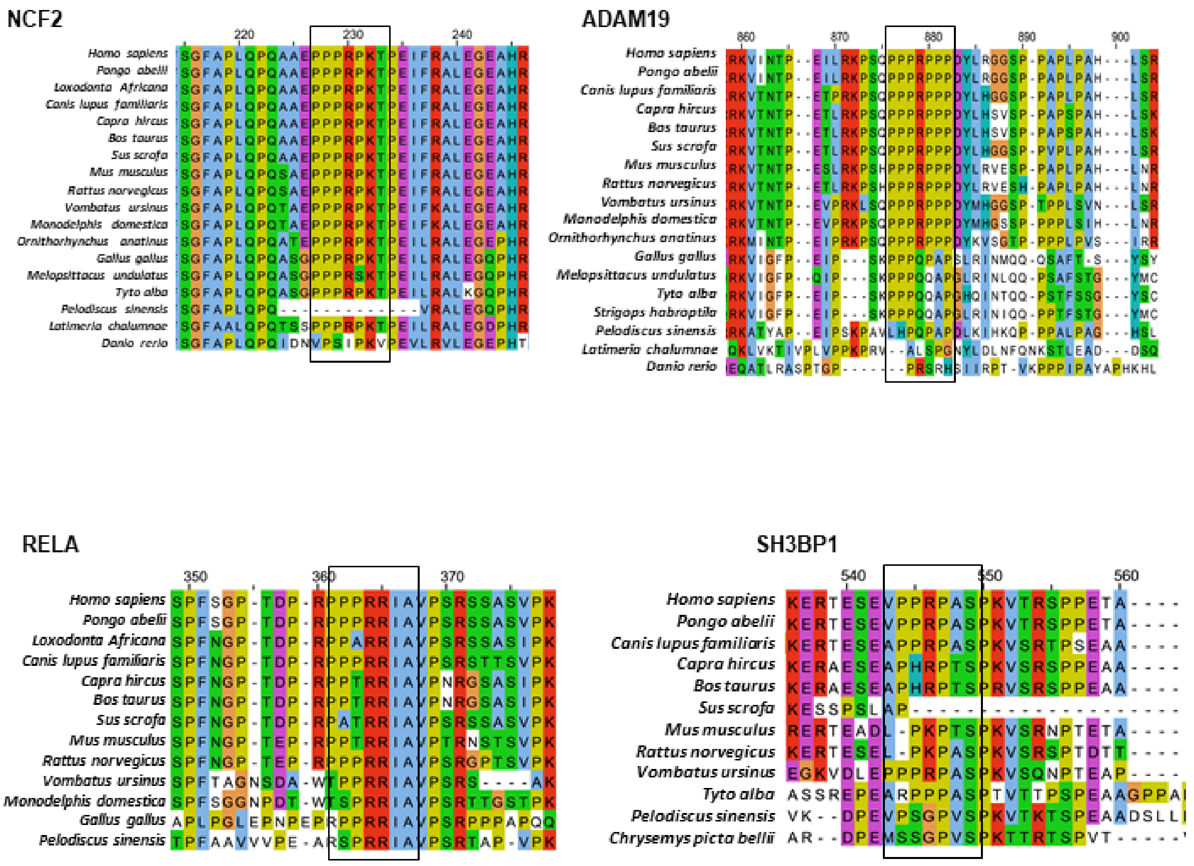

Figure S5: Sequence alignments of the proposed motifs within binding partners from Table 2 on vertebrate species. Only the proximity of the motifs are depicted and the motifs themselves are boxed.

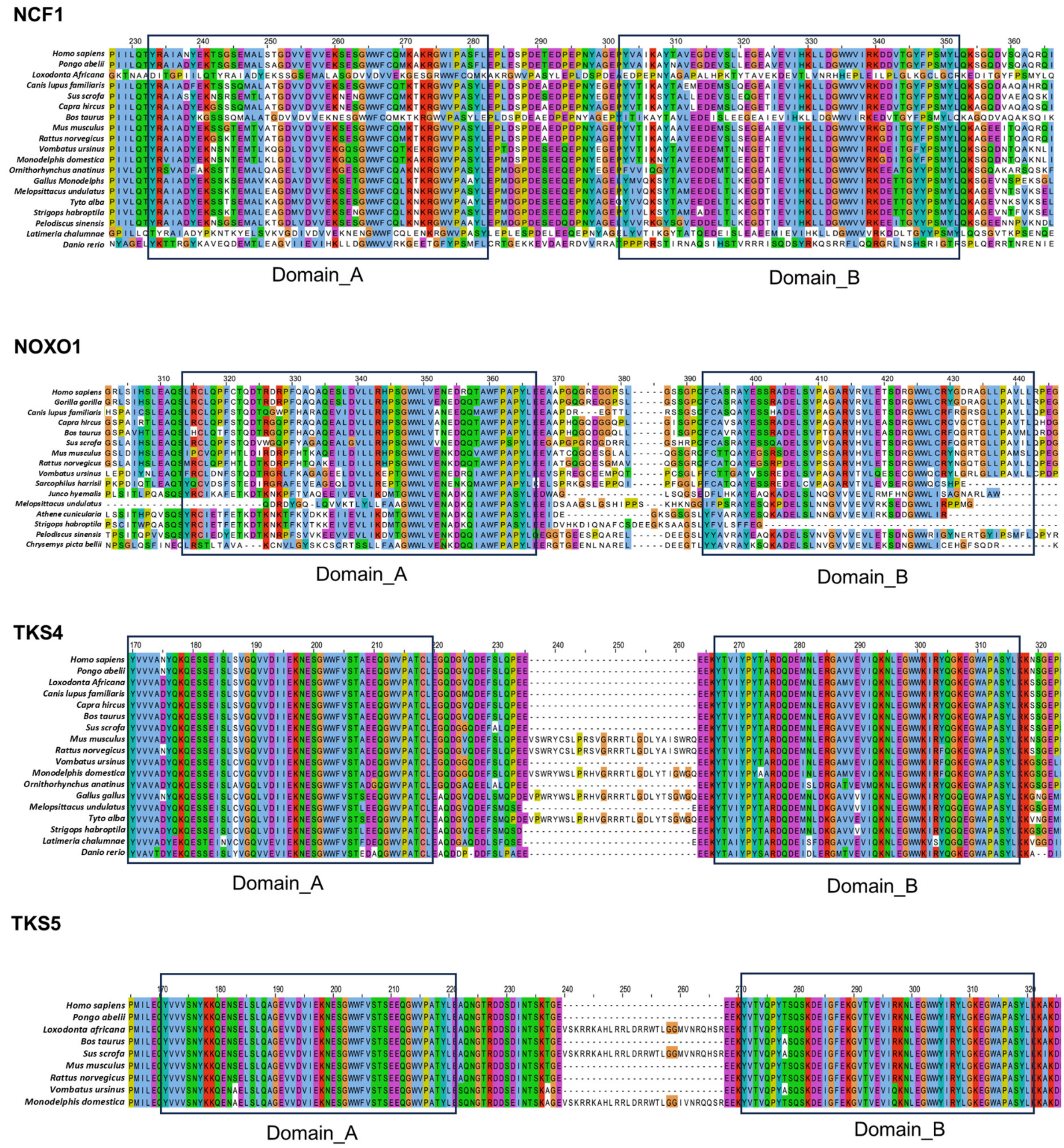

Figure S6: Sequence alignments of the tandem SH3 domains of NCF1 family members. Domain boundaries of SH3 domains are highlighted.

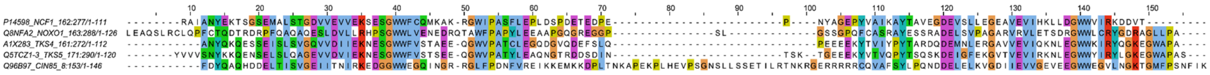

Figure S7: Sequence alignment of the tandem SH3 domains in NCF1 family members and CIN85. The alignment shows that while the GWW, C-double negative (C-[DE][DE]) and N-PxxΦL tandemization and tSH3 motif binding signatures are well-conserved in CIN85, the linker is much longer and oppositely charged compared to NFC1 family members.

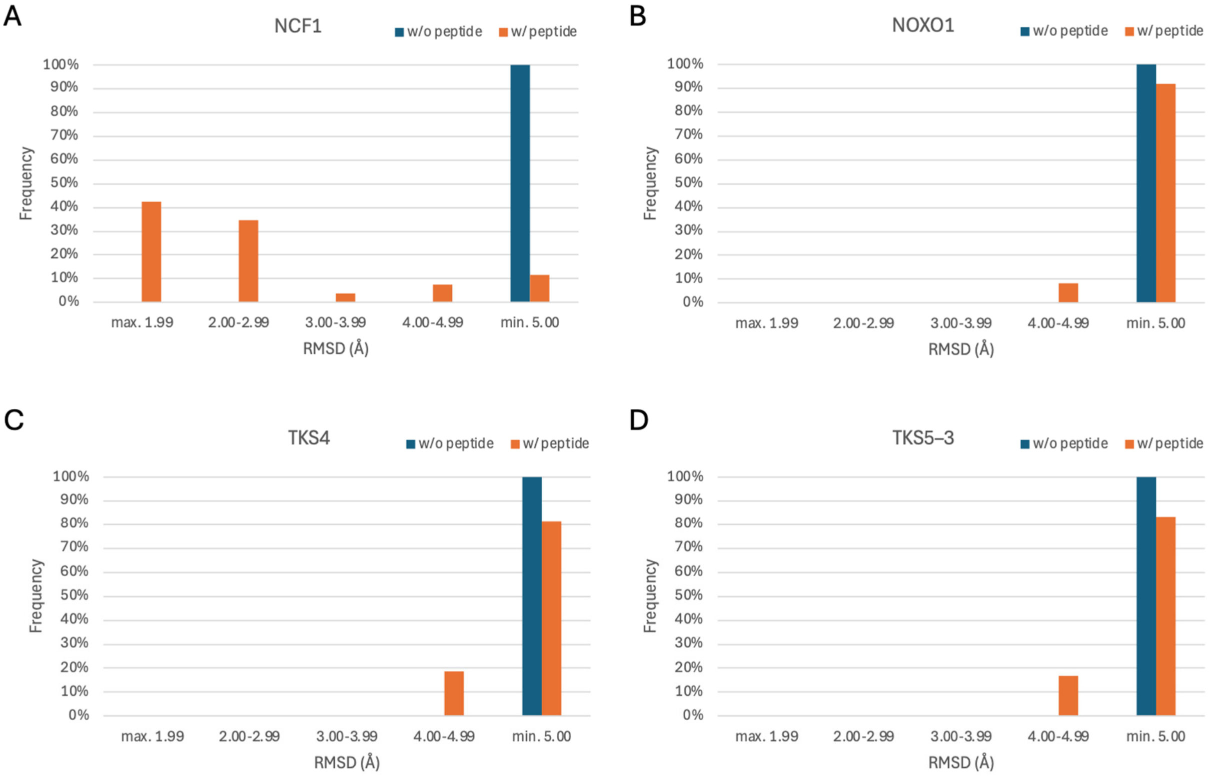

Figure S8: Structural superposition statistics of the BioEmu predicted ensembles with the reference state of tandem SH3 domain. Structural superpositions of (A) NCF1, (B) NOXO1, (C) TKS4 and (D) TKS5–3 ensemble conformers with the chosen reference structure of NCF1 tandem SH3 solved by X-ray crystallography at resolution of 2.5Å.
